# Evidence for Occurrence, Persistence, and Growth Potential of *Escherichia coli* and Enterococci in Hawaii’s Soil Environments

**DOI:** 10.1264/jsme2.ME11305

**Published:** 2011-12-06

**Authors:** Muruleedhara N. Byappanahalli, Bruce M. Roll, Roger S. Fujioka

**Affiliations:** 1 U. S. Geological Survey, Great Lakes Science Center, Lake Michigan Ecological Research Station, Porter, Indiana 46304; 2 University of Hawaii at Manoa, Water Resources Research Center, 2540 Dole Street, Holmes Hall 283, Honolulu, Hawaii 96822

**Keywords:** autochthonous population, fecal indicator bacteria, nonpoint source contamination, soil microbiota, recreational water quality

## Abstract

High densities of *Escherichia coli* and enterococci are common in freshwaters on Oahu and other Hawaiian Islands. Soil along stream banks has long been suspected as the likely source of these bacteria; however, the extent of their occurrence and distribution in a wide range of soils remained unknown until the current investigation. Soil samples representing the seven major soil associations were collected on the island of Oahu and analyzed for fecal coliforms, *E. coli*, and enterococci by the most probable number method. Fecal coliforms, *E. coli*, and enterococci were found in most of the samples analyzed; log mean densities (MPN ± SE g soil^−1^) were 1.96±0.18, *n*=61; 1.21±0.17, *n*=57; and 2.99±0.12, *n*=62, respectively. Representative, presumptive cultures of *E. coli* and enterococci collected from the various soils were identified and further speciated using the API scheme; at least six species of *Enterococcus*, including *Enterococcus faecalis* and *Enterococcus faecium*, were identified. In mesocosm studies, *E. coli* and enterococci increased by 100-fold in 4 days, after mixing sewage-spiked soil (one part) with autoclaved soil (nine parts). *E. coli* remained metabolically active in the soil and readily responded to nutrients, as evidenced by increased dehydrogenase activity. Collectively, these findings indicate that populations of *E. coli* and enterococci are part of the natural soil microflora, potentially influencing the quality of nearby water bodies.

High densities of the fecal indicator bacteria (FIB), *Escherichia coli* and enterococci, with counts often exceeding the allowable standard for recreational freshwaters (*e.g.*, geometric mean 126 colony-forming units, CFU 100 mL^−1^, *E. coli*; 35 CFU 100 mL^−1^, enterococci), are common in the watersheds of tropical islands, such as Hawaii, Guam, and Puerto Rico (15, 17, 22). Numerous sanitary surveys conducted over the years have suggested that these bacteria were likely derived from environmental sources unrelated to fecal contamination since high FIB densities were rather common in relatively pristine waters (5, 17, 35). Early investigations indicated that stream bank/riparian soils were the likely source of the indicator bacteria in the water, introduced by runoff and related hydrological processes (15, 21). Subsequent studies have confirmed the occurrence of *E. coli* in soils across geographical locations with comparable biomes: in particular, Puerto Rico (27) and south Florida (12, 39). It should, however, be emphasized that many of these studies have focused on soils around stream or river banks; thus, the extent of FIB occurrence, distribution, and growth potential in a wide range of soils types remains little understood.

The main objectives of the current investigation were to (a) determine the prevalence and relative distribution of FIB (fecal coliform, *E. coli*, and enterococci) in different soil groups (referred to as soil associations henceforth) on the island of Oahu, Hawaii, (b) identify and confirm the existence in soil of *E. coli* and enterococci bacteria using traditional cultural and biochemical methods, and (c) determine whether *E. coli* and enterococci have the ability to persist and grow in the soil environment. To our knowledge, the current study is the first detailed investigation on the occurrence, distribution, and characterization of *E. coli* and enterococci in natural, tropical soil environments.

## Materials and Methods

### Sampling sites

**Fig. 1:**
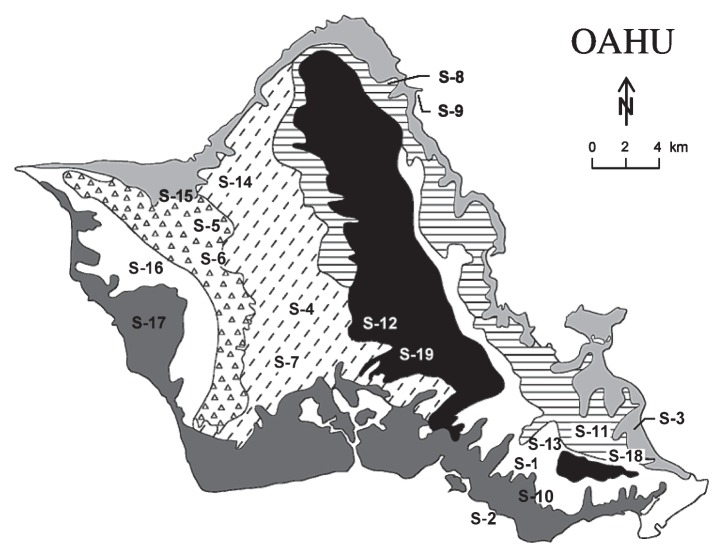
Map of Oahu showing the approximate locations where soil samples representing the seven different soil associations were collected. Location names (denoted by S-1 to S-19 on the map) and the general characteristics of the soil associations are provided in Table 1 and Table 2, respectively. Soil associations are shown by the following symbols: (1) Lualualei-Fill land-Ewa association, 
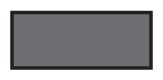
 (2) Halemana-Wahiawa association, 
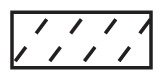
 (3) Tropohumults-Dystrandepts association, 
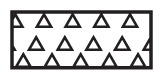
 (4) Rough mountainous land-Kapaa association, 
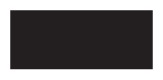
 (5) Rock land-Stony steep land association, 
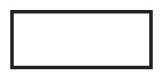
 (6) Kaena-Waialua association, 
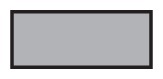
 and (7) Lalekaa-Waikane association, 
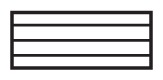

Soil samples were collected between 1992 and 1998. Samples for FIB occurrence in the various soils on the island of Oahu, Hawaii were collected in 1992; in certain locations, additional samples were collected again between 1997 and 1998. In all, samples were collected from 19 different locations (S-1 to S-19), representing the seven major soil associations found on Oahu (Fig. 1). While many of the soil affiliations on Oahu have been revised (*i.e.*, reclassified) as soil orders, for illustration purposes we have used the old classification system (*i.e.*, soil association) in this manuscript. The general characteristics of these soil associations and the sampling location names are provided in Tables 1 and 2, respectively.

Samples of surface (0–6 cm depth for routine microbiological analyses) and sub-surface (6–20 cm depth for mesocosm studies) soil were collected, transferred into sterile bottles or plastic bags, and transported to the laboratory in a cooled ice chest. Unless otherwise indicated, all samples were stored at 4°C and analyzed within 24 h of collection.

### Microbiological analyses

The general protocol for bacterial elutriation from soil is provided elsewhere (8). Briefly, an aliquot (usually 10 g) of well-mixed, fresh soil was placed in a dilution bottle containing 95 mL phosphate-buffered water, PBW (prepared from stock solutions of KH_2_PO_4_ [34 g L^−1^] and MgCl_2_·6H_2_O [81.1 g L^−1^]; 1.25 mL KH_2_PO_4_ and 5.0 mL MgCl_2_ stock solutions were used to prepare the diluent PBW solution [1000 mL with a final pH 7.0±0.2]). The soil-PBW mixture was vigorously shaken for 15 min on a wrist action shaker and the suspension was left to stand for a few minutes and then serially diluted (10-fold) as necessary; 3–5 dilutions, typically from 10^−1^–10^−5^, were used to analyze fecal coliforms, *E. coli*, and enterococci by the 5-tube most probable number (MPN) method.

Detailed procedures for isolating, identifying, and enumerating fecal coliforms, *E. coli*, and enterococci by the MPN method are available elsewhere (3). Briefly, for fecal coliforms (including *E.coli*), the soil dilutions were first inoculated into tubes containing lauryl tryptose broth and incubated at 35°C for 24–48 h; a loopful of growth from positive tubes (*i.e.*, acid growth or gas) was then transferred into tubes containing EC broth and/or EC broth plus 4-methylumbelliferyl-β-D-glucuronide (MUG). Tubes showing gas and/or acid growth (EC broth) or gas and/or acid growth plus fluorescence (EC broth plus MUG) were scored as positive tests for fecal coliforms and *E. coli*, respectively, and the corresponding bacterial counts were calculated using published MPN tables (51).

For enterococci, the soil dilutions were initially inoculated into tubes containing azide dextrose broth. Bacterial growth from turbid tubes was then streaked onto Pfizer selective *Enterococcus* agar and the plates were incubated at 35°C for 24–48 h. Brownish-black colonies (*i.e.*, fecal streptococci) were confirmed as enterococci by positive growth in brain–heart infusion (BHI) broth at 45°C and in BHI +6.5% NaCl at 35°C; enterococci counts were calculated using MPN tables. Counts of fecal coliforms, *E. coli*, and enterococci are expressed as MPN g soil^−1^.

### Identification of *E. coli* and enterococci

Pure cultures of presumptive *E. coli* were confirmed by the API 20E (bioMérieux, Hazelwood, MO, USA) identification system. Prior to speciation, all presumptive enterococci isolates were confirmed by recommended tests, such as catalase reaction and esculin hydrolysis (3); additionally, growth on mE agar (2) and reaction to Lancefield group D antigen (2) were tested on representative isolates using appropriate control cultures (*e.g.*, *Enterococcus faecalis* ATCC 19433; ATCC 29212). Isolates that were positively identified as belonging to the enterococci group were speciated using the API 20 Strep identification system (bioMérieux).

### *E. coli* metabolic status in soil

Dehydrogenase activity, a traditional enzymatic assay, has been extensively used to measure the overall metabolic activity of microbial populations in soils (38). In the current study, dehydrogenase activity was used to determine whether *E. coli* introduced into a soil under controlled (i.e., laboratory) conditions would remain metabolically active, a requirement for cell replication. Cobalt-irradiated (dose 1,750 kilorad for 33 h) Waimanalo soil (200 g) was supplemented with 2 g calcium carbonate (CaCO_3_; to buffer soil against pH changes), and the contents were thoroughly mixed. Chemical characteristics, such as pH, percent organic carbon and nitrogen, as well as concentrations of other inorganic nutrients (phosphorus, potassium, calcium, and magnesium; mg kg soil^−1^) of the fresh Waimanalo soil, are shown in supplemental Table S1. A 6-g portion of this mixture was transferred to each of 12 test tubes (16×150 mm) representing various treatments and corresponding controls, each replicated twice as follows: (i) uninoculated control, (ii) uninoculated control + peptone (1 g 100 g soil^−1^), (iii) soil + *E. coli*, (iv) soil + *E. coli* + peptone (1 g 100 g soil^−1^), (v) soil + *E. coli* + peptone (2 g 100 g soil^−1^), and (vi) natural Waimanalo soil + peptone (1 g 100 g soil^−1^; positive control).

A pure culture of *E. coli* (ATCC 25922) was grown overnight in tryptic soy broth. Cells were thoroughly washed with PBW (2×) to remove excess nutrients and the inoculum was adjusted to the desired cell density using PBW as the diluent. The soil in 6 test tubes was inoculated with *E. coli* at a target concentration of about 1.48×10^8^ cells g dry soil^−1^. For comparison, a natural Waimanalo soil containing indigenous microbial populations and amended with peptone (positive control) was placed in 2 tubes. To each tube, 1 mL of 3% aqueous solution of 2,3,5-triphenyltetrazolium chloride, TTC (Sigma-Aldrich, St. Louis, MO, USA) and 2.5 mL distilled water was added, and then the contents of each tube were mixed thoroughly and stoppered with a cap. The tubes were then transferred to an anaerobic jar and incubated at 37°C for 48 h. The extraction of triphenyl formazan (TPF) from the soil and measurement of its concentration, an indicator of dehydrogenase activity, were performed according to procedures outlined in the Methods of Soil Analysis, Part 2: Microbiological and Biochemical Properties (44). Dehydrogenase activity was expressed as μg formazan g dry soil^−1^.

### Growth potential of *E. coli* and enterococci

A pilot study was conducted to find out whether *E. coli* and enterococci from contaminated soil could grow when introduced into a new soil devoid of indigenous microflora. One part (25 g) of sewage-spiked soil (from an earlier experiment) was mixed with nine parts (225 g) of autoclaved stream bank soil collected from the banks of Manoa Stream near the University of Hawaii; *E. coli* and enterococci densities (log MPN g dry soil^−1^) in the sewage-contaminated soil were 1.03×10^3^ and 6.78×10^4^, respectively. The soil was then brought to about 65% of maximum water-holding capacity and maintained at that level throughout the experiment (~25°C). At regular intervals, subsamples were analyzed for *E. coli* and enterococci by the MPN method, as previously described.

### Statistical analyses

Statistical analyses were performed using SPSS version 12.0 (41). Statistical procedures were performed on log_10_-transformed data to meet parametric assumptions of equality of variance and normal distribution. Non-parametric tests (*e.g.*, Kolmogorov-Smirnov test) were used to test normality. Analysis of variance was used to compare means; unless otherwise stated, statistical significance was set at α=0.05.

## Results

### FIB occurrence in major soil associations on Oahu

**Fig. 2:**
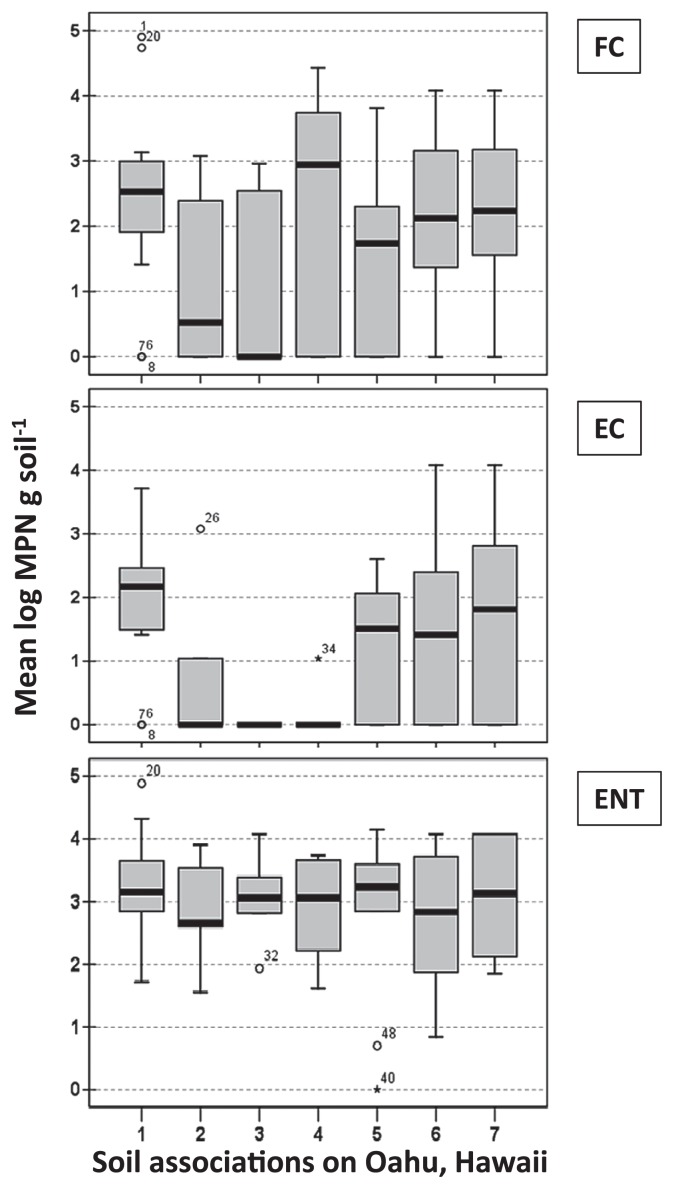
Mean log densities of fecal coliforms (FC; top panel), *E. coli* (EC; middle panel), and enterococci (ENT; bottom panel) in soil samples for the seven major soil associations. Black bars, medians; rectangles, 25^th^ and 75^th^ percentiles; thin bars, range (excluding outliers); open circles, outliers; asterisk, samples with counts below detection limit (4 MPN g soil^−1^). Numbers 1–7 designate soil associations (additional details are provided in Table 2): 1, Lualualei-Fill land-Ewa association; 2, Halemana-Wahiawa association; 3, Tropohumults-Dystrandepts association; 4, Rough mountainous land-Kapaa association; 5, Rock land-Stony steep land association; 6, Kaena-Waialua association; 7, Lalekaa-Waikane association.

Between 57 and 62 soil samples representing the seven major soil associations (Table 1) were analyzed for fecal coliforms, *E. coli*, and enterococci. The overall bacterial densities (mean log MPN g soil^−1^ ± SE) were 1.96±0.18 (*n*=61), 1.21±0.17 (*n*=57), and 2.99±0.12 (*n*=62) for fecal coliforms, *E. coli*, and enterococci, respectively. *E. coli* densities among the various soil associations were significantly different (*F_6, 50_*=*3.105*, *P*=*0.012*); however, no significant differences in bacterial densities were observed for fecal coliforms (*F_6, 60_*=*1.246*, *P*=*0.298*) or enterococci (*F_6, 61_0.467*, *P*=*0.829*). Variations in bacterial densities were markedly high both within a soil association and among associations (Fig. 2). In general, enterococci were recovered far more consistently than fecal coliforms and *E. coli*: 98% occurrence vs 74% and 54% occurrences, respectively.

### Confirming the identity of *E. coli* and enterococci recovered from soil samples

A total of 201 presumptive *E. coli* isolates representing the seven soil associations were confirmed as *E. coli* on the basis of biochemical reactions using API 20E miniature test panels (16). These isolates were all typed as *E. coli*, with identification grades ranging from very good to excellent.

For enterococci, the presumptive isolates were confirmed as those belonging to the enterococci group by their distinct characteristics: Gram-positive coccoid cells, negative catalase reaction, positive esculin hydrolysis, and ability to grow in BHI broth at 45°C and in BHI broth containing 6.5% NaCl at 35°C, as well as the presence of Lancefield group D antigen. Of the 68 isolates screened for confirmatory tests, at least 47 (69%) isolates were identified with certainty as belonging to the enterococci group.

Forty-seven presumptive enterococci isolates were speciated using the API 20 Strep identification scheme; additional confirmatory tests (*e.g.*, motility, presence and color of pigmentation) were necessary to confirm whether some of the isolates identified as *Enterococcus faecium* 2 or *E. faecium* 3 were likely *Enterococcus casseliflavus* (motility +, yellow pigmentation +) or *Enterococcus gallinarum* (motility +, yellow pigmentation −). Of the 47 isolates, 39 (83%) were identified as one of six species of *Enterococcus: Enterococcus avium* (1 isolate), *E. casseliflavus* (16 isolates), *Enterococcus durans* (4 isolates), *E. faecalis* (9 isolates), *E. faecium* (1 isolate) and *E. gallinarum* (8 isolates). Of the remaining eight isolates, four were typed as *Streptococcus lactis* subsp. *diacetylactis*, one as *Aerococcus viridans*, and the remaining three yielded no identification because of ambiguous biochemical profiles.

### *E. coli* metabolic status in soil

Negative controls (cobalt-irradiated soil amended or with not addition of peptone and devoid of microflora) revealed no measurable dehydrogenase activity, expressed as μg formazan g dry soil^−1^ 48 h^−1^. As a positive control, we analyzed natural Waimanalo soil with all its indigenous microflora plus peptone amendment. Maximum dehydrogenase activity (1,032 μg formazan) was observed in this soil (Table 3). In contrast, there was a steady increase in the mean dehydrogenase activity in inoculated soils, with higher activity noticeable in peptone-amended soils: *E. coli*, unamended (93 μg formazan), *E. coli* + 1 g peptone (243 μg formazan), and *E. coli* + 2 g peptone (295 μg formazan). These results provide direct enzymatic evidence that *E. coli* can remain metabolically active and potentially replicate in soil under certain conditions.

### Growth potential

Mixing one part of sewage-contaminated soil with nine parts of cobalt-sterilized soil resulted in *E. coli* and enterococci densities of 0.97 and 2.98 log MPN g dry soil^−1^ at t_0_ (Table 4). *E. coli* steadily increased over the 4-day incubation, reaching 3.12 log MPN g dry soil^−1^; likewise, enterococci counts increased to 4.58 log MPN g dry soil^−1^ during this period. The overall increase in densities was in excess of 100-fold for *E. coli* and nearly 100-fold for enterococci.

## Discussion

### FIB occurrence and distribution in soils

Over the years, reports on FIB occurrence in soil environments have primarily focused on pasture lands and agricultural soils impacted by farm animals, and studies have shown that runoff from farm lands could elevate FIB densities in nearby watersheds (14, 43, 45). There have been sporadic reports of FIB occurrence in undisturbed soils (19, 46); however, detailed investigations of their widespread occurrence and distribution across different soil types, as for those reported here, are rather limited.

The findings that FIB are common inhabitants of most of the samples analyzed (percent frequency of 74, 54, and 98 for fecal coliforms, *E. coli*, and enterococci, respectively) indicate that they are not just confined to moist stream or river bank soils on which many of the previous studies have focused (21, 36). At the same time, a clear relationship between soil associations and FIB densities was not evident in this study, suggesting that more intensive surveys are needed to determine if there is any such relationship. In our study, high variation in bacterial densities was common both within and among soil associations (see Fig. 2). Such high variation in *E. coli* counts in soils is not uncommon and has been demonstrated in a number of studies (6, 9, 25, 27), which can be explained, in part, by the microbial patchiness in these environments (32, 50). Additionally, a variety of physical, chemical, and biological factors may have affected FIB occurrence in the soils examined, as has been identified in other studies, including available moisture (6, 10, 39), competition for nutrients (7), and predation (34, 40).

Significantly, many recent studies have expanded the known area of FIB occurrence in soil beyond tropical/subtropical biomes. *E. coli* occurrence and persistence have been repeatedly demonstrated in the riparian soils of southern Lake Michigan and Lake Superior watersheds (6, 9, 25); *E. coli* has even been recovered from snow-covered forest soils, albeit at low densities (0.69 log MPN g soil^−1^) (9), indicating that it can survive the extreme wintry conditions of the Great Lakes basin. Cumulatively, these findings suggest that populations of FIB are common in soil environments in both tropical and temperate climates. Thus, soil should be considered as an environmental source of FIB, and high densities of these bacteria in streams and rivers can be attributable to two important mechanisms, both likely mediated by hydrological processes: (a) transportation of soil-bound fecal bacteria into stream waters through runoff and/or erosion (6, 15, 21) and (b) suspension of sediment-borne bacteria in overlying water by mechanical disturbance or related processes (11, 18, 23, 37).

In the current study, enterococci were consistently recovered from the soil samples (98% frequency), often at much higher densities than fecal coliforms and *E. coli* (overall log mean ± SE 2.99±0.12, 1.96±0.18, 1.21±0.17 log MPN g^−1^, respectively). To our knowledge, such widespread occurrences of enterococci (in soils) have not been reported previously, although there have been reports of enterococci occurrence on a variety of crop species, including forage grasses (30, 31, 33).

Recent investigations have expanded FIB occurrences in non-enteric habitats beyond soils. For instance, populations of *E. coli* and/or enterococci have been isolated from pitcher plant fluids (47), aquatic vegetation, such as seaweed and submerged plants (hydrilla) (1, 4), and beach sand (24, 48, 52). Collectively, these findings support the soil results presented here and generalize the observation that populations of enterococci are commonly found in a variety of aquatic and terrestrial habitats.

### *E. coli* and enterococci identification

Most of the presumptive *E. coli* cultures recovered from various soils were typed as *E. coli*, with species ID ranging from very good to excellent grading, providing credence that these isolates were indeed *E. coli*. Most of the API 20E biochemical profiles were similar; thus, no additional characterization or grouping based on these profiles was possible. In contrast, the Biolog carbon utilization patterns did show some differences, with 44 of the 48 *E. coli* isolates clustering into five distinct groups, suggesting that the soil *E. coli* populations were metabolically diverse (16). Further, relative to the Biolog database strains, numerous (soil) *E. coli* isolates were characteristically negative in their response to the utilization of certain carbon sources, such as acetic acid and amino acids (L-aspartic acid, D-serine) (8). Whether such phenotypic markers are useful in identifying environmental *E. coli* strains is unclear, but more research is needed to better understand the nutritional requirements and metabolic diversity of *E. coli* inhabiting soil and other natural environments.

Many of the confirmed enterococci isolates could not be speciated, which probably reflects the inadequacy of the phenotypic characteristics (*e.g.*, biochemical profiles) as a tool for identifying and speciating presumptive enterococci from environmental/non-clinical sources (13, 30). It was further evident that there were distinct metabolic profiles among *Enterococcus* spp based on their carbon source utilization patterns (16). For instance, 67% of the soil *E. faecalis* isolates utilized m-inositol as a carbon source; in contrast, the Biolog database strains did not use this as a carbon source (8). Whether m-inositol would be a useful marker to differentiate soil *E. faecalis* strains from other *E. faecalis* strains should be further investigated. Collectively, these results demonstrate that the soil environment of Hawaii is conducive to the occurrence of different *Enterococcus* spp. Further, our findings that *Enterococcus* spp. comprised *E. faecalis* (isolated from soil associations 5 and 7) and *E. faecium* (isolated from soil association 7) is significant since these species are frequently found in the feces of humans and warm-blooded animals and are increasingly recognized as opportunistic pathogens.

### *E. coli* metabolic status in soil

These results provide direct enzymatic evidence that the metabolic activity of *E. coli* increases as the cells respond to nutrients that apparently translate into increased cell numbers, as observed in the growth experiment (see Table 4). The dehydrogenase activity of *E. coli* in peptone-amended soil was about 3-fold higher than in the control (*i.e.*, unamended soil), but lower than that measured in natural Waimanalo soil. These results were not surprising since the higher dehydrogenase activity in natural Waimanalo soil was the cumulative response of all active microbial populations.

Under natural conditions, indigenous microflora, which are numerous and better adapted to soil conditions, might outcompete *E. coli* for available resources and thus limit its growth; however, under certain conditions, such as reduced competition or when nutrients are in excess, *E. coli* can grow in the presence of other organisms (7, 49). Taken together, these findings demonstrate that *E. coli* can remain metabolically active and has the potential to grow in the soil under natural conditions.

### *E. coli* and enterococci growth potential

The ability to colonize a new habitat is critical for microbial survival in that habitat. This is particularly essential for *E. coli* and enterococci, which have often been considered as transient organisms in heterothermic environments. In the current study, both *E. coli* and enterococci readily established in new, autoclaved soil; both of these bacteria grew about 100-fold in 4 days. The rapid increase of these bacteria was likely due, in part, to the reduced competition for available nutrients, as previously shown in studies by Byappanahalli and Fujioka (7) and Whitman *et al.*(49). We recognize that these results are preliminary and do not fully explain the *E. coli* enterococci ubiquity in the soils on Oahu. At the same time, it is important to identify that soil colonization by FIB is a cyclical process, with bacteria being introduced or reintroduced by one or several sources, such as from adjacent soil, animal feces, dust particles, insects, and runoff. Additional studies are needed to better elucidate these ecological processes.

It is clear that *E. coli* and enterococci represent a small fraction of the soil microflora; however, their ubiquity in this environment suggests an autochthonous population independent of anthropogenic inputs (9, 25). Further, recent evidence corroborating the occurrence of environmentally adapted *E. coli* clades (29) strongly supports this observation. While the original source of soil-borne *E. coli* and enterococci currently remains speculative, bacterial inputs from these sources to nearby water bodies through erosion and runoff can adversely affect the microbiological quality of such impacted waters. Moreover, once these bacteria are transported and deposited onto sediments, they can survive much longer in these environments than in water columns (18, 20). Hydrometeorological events, such as increased flow, run off, and high waves (18, 26, 28, 42), could in turn resuspend sediment-borne FIB and increase their densities, potentially influencing the water quality.

## Conclusions

Through a series of field and lab investigations, evidence was obtained to show that populations of FIB are naturally present in high densities in the soil environments of Oahu, Hawaii. The major findings were: (i) representative soils of the seven major associations harbored fecal coliforms (including *E. coli*) and enterococci bacteria, with high variation in their densities both within and across soil associations, (ii) various biochemical tests confirmed the presence of *E. coli* and enterococci in the soil environments; at least six different species of *Enterococcus*, including *E. faecalis* and *E. faecium*, were recovered from soils, (iii) *E. coli* and enterococci had the ability to colonize the soil environment, and (iv) *E. coli* remained metabolically active, an important ecological requirement for its persistence in the soil environment.

To our knowledge, the current study is the first documentation of the widespread occurrence of FIB across different soil types on the island of Oahu. These findings strongly support the idea that soils should be considered as an environmental source of FIB in Hawaii and other tropical and temperate biomes elsewhere. At the same time, many ecological questions relating to their occurrence in soil environments remain unclear. For instance, (a) what is the original source of these bacteria? (b) How are these bacteria able to colonize, adapt, and grow in soil conditions? (c) Are soil FIB populations genetically different from their counterparts in human and animal gastrointestinal tracts? A greater understanding of these processes is critical for identifying soil as a habitat for FIB and to explore whether these bacteria have any secondary roles in these environments.

## Supplementary material



## Figures and Tables

**Table 1:** Sampling locations: location codes, S-1 to S-19 in parentheses, correspond to those marked on the map of Oahu in Fig. 1

I. University Hawaii campus (S-1)
Banks of Manoa Stream
East-West Road (Holmes Hall)
East-West Road (Kennedy Theatre)
East-West Road (UH Health Center)
Maile Way (St. John’s Hall)
Correa Road (Geophysics Building)
Correa Road (Campus Book Store)
II. Recreational areas (S-2)
Ala Moana Beach Park
Old Stadium Park
Kapiolani Park
III. Other major sampling locations on Oahu
Bellows (S-3)
East Mililani (S-4)
Kaukonahua Road (S-5)
Kolekole Pass (S-6)
Kunia Road (S-7)
Upper Laie (S-8)
Lower Laie (S-9)
Lower Manoa (S-10)
Olomana (S-11)
Pacific Palisades (S-12)
Upper Manoa (S-13)
Waialua (S-14 and S-15)
Waianae Kai (S-16)
Waianae Valley (S-17)
Waimanalo (S-18)
Waimano (S-19)

**Table 2:** Soil associations on the island of Oahu, Hawaii and their general characteristics

Soil association	Symbol	General characteristics
1	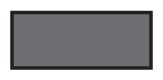	**Lualualei-Fill land-Ewa association:** Deep, nearly level to moderately sloping, well-drained soils that have a fine textured or moderately fine textured subsoil or underlying material, and areas of fill land; on coastal plains.
2	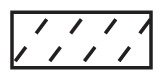	**Halemana-Wahiawa association:** Deep, nearly level to moderately sloping, well-drained soils that have fine-textured subsoil; on uplands.
3	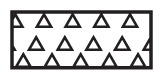	**Tropohumults-Dystrandepts association:** Gently sloping to very steep, well-drained soils that are underlain by soft weathered rock, volcanic ash, or colluvium; on narrow ridges and side slopes.
4	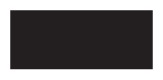	**Rough mountainous land-Kapaa association:** Very steep land broken by numerous drainage-ways and deep, well-drained soils that have fine textured or moderately fine textured subsoil; in gulches and on narrow ridges.
5	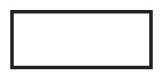	**Rock land-Stony steep land association:** Steep to precipitous, well-drained to excessively drained, rocky and stony land.
6	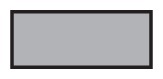	**Kaena-Waialua association:** Deep, mainly nearly level and gently sloping poorly drained to excessively drained soils that have a fine-textured to coarse-textured subsoil or underlying material; on coastal plains and talus slopes and in drainageways.
7	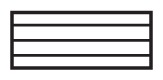	**Lalekaa-Waikane association:** Deep, nearly level to very steep, well-drained soils that have dominantly fine-textured subsoil; on fans, terraces, and uplands.

Source: USDA Soil Conservation Service and the University of Hawaii Agricultural Experiment Station (August 1972)

**Table 3:** *E. coli* metabolic status in cobalt-irradiated Waimanalo soil (CI) amended or unamended with nutrients as determined by dehydrogenase activity

Treatments	μg formazan g dry soil^−1^ 48 hr^−1^ (mean ± standard deviation)
CI soil (negative control)	00
CI soil + peptone^a^ (negative control)	00
CI soil + *E. coli*	93.0±0.0
CI soil + *E. coli* + peptone^a^	243.2±16.3
CI soil + *E. coli* + peptone^b^	295.4±24.6
Natural soil + peptone^#^ (positive control)	1032.0±45.6

Negative controls=cobalt-irradiated Waimanalo soil devoid of indigenous microbes

No measurable formazan (00) was detected in negative controls Positive control=natural Waimanalo soil containing indigenous microbes

a 1 g peptone100 g soil^−1^;

b 2 g peptone 100 g soil^−1^*E. coli* was added at the rate of 1.48×10^8^ cells g dry soil^−1^

**Table 4:** *E. coli* and enterococci growth potential in soil, after mixing one part of sewage-spiked soil with nine parts of sterile (autoclaved) soil

Time (days)	Bacterial densities (log MPN g dry soil^−1^)	

	*E. coli*	enterococci
0	0.97	2.78
1	2.36	ND
2	2.82	ND
3	2.97	4.47
4	3.12	4.58

ND=not determined.
