# Supplementary material for: Evidence for Occurrence, Persistence, and Growth Potential of Escherichia coli and Enterococci in Hawaii’s Soil Environments
Source: Microbes Environ. 2011 Dec 6;27(2):164–70. doi: 10.1264/jsme2.ME11305 (PMC4036009; doi:10.1264/jsme2.ME11305)
Supplement: Supplementary file 1 [file 27_164_s1.pdf]

Table S1. Chemical characteristics of the fresh, untreated subsurface Waimanalo Soil collected at the Waimanalo Experimental Station, University of Hawaii.

pH	Percent organic carbon	Percent nitrogen	Other inorganic nutrients (mg kg^{-1})			
			Phosphorus	Potassium	Calcium	Magnesium
6.2	1.75	0.17	66	320	3700	980
